# Three cases of Zika virus imported in Italy: need for a clinical awareness and evidence-based knowledge

**DOI:** 10.1186/s12879-016-1973-5

**Published:** 2016-11-11

**Authors:** Emanuele Nicastri, Raffaella Pisapia, Angela Corpolongo, Francesco Maria Fusco, Stefania Cicalini, Paola Scognamiglio, Concetta Castilletti, Licia Bordi, Antonino Di Caro, Maria Rosaria Capobianchi, Vincenzo Puro, Giuseppe Ippolito

**Affiliations:** 1 National Institute for Infectious Diseases Lazzaro Spallanzani-IRCCS, Via Portuense, 292- 00149 Rome, Italy; 2 Istituto Nazionale per le Malattie Infettive “Lazzaro Spallanzani”, Via Portuense 292, 00149 Rome, Italy

**Keywords:** Zika virus, Emerging or re-emerging diseases, Imported viral diseases, Pregnancy, Surveillance, Travel

## Abstract

**Background:**

Since early 2015, a large epidemic of Zika Virus (ZIKV) is spreading across South and Central America. An association between congenital neurological malformations (mainly microcephaly), other neurological manifestations such as Guillain-Barrè Syndrome, and ZIKV infection is suspected.

**Case presentation:**

Three confirmed cases of ZIKV in travelers returning from Brazil between May 2015 and January 2016 are described. All patients had mild symptoms with no neurological complications.

**Conclusions:**

An increasing awareness among clinicians about this emerging disease is advisable, both for the need to provide correct additional information to the patients and to travelers, with a special focus on pregnant women, and for the presence of the competent vector in Southern Europe.

**Electronic supplementary material:**

The online version of this article (doi:10.1186/s12879-016-1973-5) contains supplementary material, which is available to authorized users.

## Background

In May 2015, the first report of autochthonous Zika Virus (ZIKV) infection has been confirmed in Brazil. The ZIKV epidemic is rapidly evolving [1, 2] and autochthonous transmission has been also reported out of Latin American Countries [1, 2]. At the same time, several imported cases have been described in USA and European countries [1, 2].

ZIKV disease is caused by a virus of the *Flaviviridae* family transmitted to humans by *Aedes* mosquitoes. Perinatal transmission has been reported, as well as human-to-human transmission through blood transfusion and sexual intercourse [3–5].

The main clinical manifestations of ZIKV are low grade fever (<38.5 ° C), maculopapular rash, headache, conjunctivitis, myalgia and arthralgia, with possible small joint swelling. The clinical course is generally lasting 2–7 days, and, as for other flavivirus infections, a high rate of asymptomatic infection with ZIKV is expected [2]. Most people full recover without severe complications, and hospitalisation rate is low, but fatal outcome has been described in patients with underlying diseases [6].

Furthermore, an unusual increase of congenital malformation, in particular microcephaly, and Guillain–Barré syndrome cases, have been reported in areas where ZIKV epidemic is ongoing. An association with ZIKV epidemic has been strongly suspected and is under investigation [2].

As the epidemic will continue to extend, an increasing number of imported cases are expected. In Italy, the risk for an autochthonous transmission cannot be excluded, since the competent vector (*Aedes albopictus*) is present and it has already caused a large outbreak of Chikungunya (CHIKV) disease in 2007 [7, 8].

We describe three cases of imported ZIKV fever to Italy, in travelers returning from Brazil, observed at the Lazzaro Spallanzani National Institute for Infectious Diseases (INMI), Rome.

This manuscript adheres to CARE guidelines/methodology [9], and the CARE checklist is reported in Additional file 1.

## Case presentation

### Patient 1

A 74-year-old Italian man sought medical attention, as outpatient, on May 2015, two days after returning from Fortaleza, North-East of Brazil, where he spent the last 5 months. He reported six days of maculopapular rash, low-grade fever, significant joint and muscle tenderness, malaise and bilateral wrists swelling. At the physical examination, he showed a papular rash spreading to extremities including the palms. Neurological examination was normal. All symptoms completely resolved within few days. The presence of rash guided the laboratory investigations, and serology for HIV, syphilis, *C. psittaci*, *M. pneumoniae*, CHIKV and Dengue Virus resulted negative for acute infection. Widal serodiagnosis, Quantiferon, and PCR, in urine specimen, for both *N. gonorrhea* and *C. trachomatis* resulted negative. An indirect immnofluorescence assay was positive for ZIKV: 1:40 IgM and 1:80 IgG titers, RT-PCR for *Flavivirus* genus both in serum and urine and RT-PCR for Chikungunya in serum sample were negative. A follow up sample at day 39 after symptoms onset, showed a 4-fold increase of the anti ZIKV antibodies titer (1: 160 IgM and 1:320 IgG) and at day 56 the anti ZIKV antibodies titer was 1:80 IgM and > =1:1280 (see Table 1). The patient fully recovered with no complications.

**Table 1:** Diagnostic data from 3 patients with ZIKV at INMI, Rome [17–20]

		Patient 1		Patient 2		Patient 3	
		At diagnosis (7)*	At day 32 (39)*	At diagnosis (2)*	At day 12 (14)*	At diagnosis (3)*	Ad day 7 (11)*
ZIKV	RT-PCR serum^a^	Negative	Not performed	Positive	Not performed	Positive	Negative
	RT-PCR urine^a^	Negative	Not performed	Positive	Not performed	Positive	Positive
	IgM^b^	1:40	1:160	1:40	1:640	<1:20	1:80
	IgG^b^	1:80	1:320	<1:20	> = 1:640	<1:20	1:160
	NT^g^	<1:20	1:160	Not Performed	<1:20	Not Performed	1:40
Dengue Virus	RT-PCR serum^c^	Negative	Not performed	Negative	Not performed	Negative	Not performed
	RT-PCR urine^c^	Not performed	Not performed	Negative	Not performed	Negative	Not performed
	IgM^d^	<1:20	Not performed	<1:20	Not performed	<1:20	Not performed
	IgG^d^	<1:20	Not performed	<1:20	Not performed	1:20	Not performed
CHIKV	RT-PCR serum^e^	Negative	Not performed	Negative	Not performed	Negative	Not performed
	IgM^f^	<1:20	Not performed	<1:20	Not performed	<1:20	Not performed
	IgG^f^	<1:20	Not performed	<1:20	Not performed	<1:20	Not performed

*Numbers in brackets indicate days from symptoms onset

^a^pan-flavivirus NS5 nested RT-PCR (modified from [17]), followed by the amplicons’ sequencing and phylogenetic analyses

^b^: IgG and IgM in house indirect immunofluorescence assay performed with homemade slides spotted with a mix (1:1) of uninfected and ZIKV (MR766 strain)–infected Vero E6 cells, according to [18]. IFA titres <1:20 were considered negative

^c^: CDC DENV-1-4 Real-Time RT-PCR Assay for Detection and Serotype Identification of Dengue Virus (http://www.cdc.gov/dengue/clinicalLab/realTime.html)

^d^: IgG and IgM indirect immunofluorescence assay Euroimmun Flavivirus Mosaic 2 slides. IFA titres <1:20 were considered negative

^e^: Real-time RT-PCR targeting the E1 structural protein [19]

^f^: IgG and IgM indirect immunofluorescence assay Euroimmun Chikungunya virus slides. IFA titres <1:20 were considered negative

^g^: Neutralisation test (NT) titres <1:20 were considered negative [20]

### Patient 2

On August 2015 a 58-year-old Italian man was admitted to the Infectious Diseases ward at INMI, because of febrile illness and maculopapular rash since two days. He spent holidays in Brazil (Rio De Janeiro) for two weeks, and returned in Italy 3 days before hospital referral. At admission, he was febrile (38.5 ° C) and physical examination showed a maculopapular rash on the trunk and upper extremities. Routine blood tests including haemogram and bio-chemical exams were all within normal ranges. Thick and thin blood smears were negative for malaria. Serology was negative (<1:20 for both IgM and IgG) for Chikungunya and Dengue viruses, an indirect immunofluorescence assay (IFA) for ZIKV revealed a 1:40 IgM titer with <1:20 IgG titer. RT-PCR for *Flavivirus* genus resulted positive both in serum and urine, the RT-PCR product was sequenced and ZIKV infection confirmed. During hospitalization the symptoms rapidly resolved and the patient was discharged at day 5. Follow up ZIKV serologic testing, one week after discharge, showed increased specific antibodies titer (1:640 IgM and > =1:640 IgG) (see Table 1). The patient fully recovered with no complications.

### Patient 3

On January 2016 a 36-year-old Italian woman presented at the Infectious Diseases outpatient department, with a widespread maculopapular rash covering the trunk, arms, and legs, started 2 days after returning from a 8-days vacation in Brazil (Rio de Janeiro and Buzios). The patient had been vaccinated against Yellow Fever. At medical examination, no other symptoms and signs were recognized. Laboratory tests showed leukopenia (leukocyte count 3.5 × 109 cells/L, range 4.00–10.80 × 10^9^ cells/L) with lymphopenia (lymphocytes 11.0 %, range 20.0–40.0 %) only. Pregnancy test was negative. An indirect immunofluorescence assay resulted negative (<1:20 for both IgM and IgG) for ZIKV and Chikungunya, while IgG titer for Dengue Virus was weakly positive, probably because of cross-reactivity with previous yellow fever vaccination. RT-PCR for *Flavivirus* genus resulted positive both in serum and urine, samples were sequenced and ZIKV was confirmed. The patient received a counseling about potential of ZIKV sexual transmission, the need to delay a possible pregnancy and appropriate methods to reduce this risk. The symptoms resolved spontaneously in one day. A follow up ZIKV serologic testing, performed at day 11 after symptoms onset was positive for both IgM (1:80) and IgG (1:160), pan-flavivirus RT-PCR was negative on serum whereas was still positive on urine (see Table 1).

For all patients, the presence of ZIKV-specific neutralising antibodies in serum samples was also confirmed by a virus neutralization assay.

## Conclusions

At our institution, since May 2015, three cases of ZIKV from Brazil were diagnosed. The recognition of this infection, in the early stage of the epidemic when only very few cases have been reported in Europe, required an high-level of clinical awareness. The inclusion of ZIKV in the evaluation for arboviral diseases is mandatory to provide and early diagnosis and a timely notifications to public health authorities. Indeed, ZIKV infection is characterized by symptoms overlapping those of Dengue and Chikungunya infections. Moreover, sometimes the presence of ZIKV antibodies may cause cross-reaction in diagnostic tests of other *flavivirus*, and thus the test interpretation require an high-level of expertise [10, 11]. INMI established serological and molecular diagnostics for ZIKV in February 2014, and included it in the diagnostic menu for fever in travelers returning from tropical countries.

This report, especially in the cases of patients 1 and 2 who were identified at the beginning of ZIKV epidemic, demonstrates that an integrated model combining clinical awareness, updated epidemiological knowledge, and advanced diagnostic methods, is able to timely identify patients affected by emerging diseases. This integrated approach has long been implemented by INMI in facing emerging and re-emerging diseases [12].

Moreover, the description of these cases is important, in order to disseminate the knowledge about this disease. Indeed, the role of clinicians in the ZIKV infections is not limited to the disease diagnosis and management, but additional information should be given to the patients. Increasing evidence supports a link between the ZIKV infection during pregnancy and the occurrence of congenital neurological malformations (mainly microcephaly). Consequently, appropriate information should be given to pregnant women who travelled in endemic area about their risk and about the correct diagnostic procedures and surveillance protocols. For the same reason, it is important to advise pregnant women, or women who are planning a pregnancy, to consider postponing travel to endemic areas [13]. Moreover, some reports strongly support the possibility of transmission through sexual intercourse and blood transfusion: therefore, it is also important to inform travellers returning home from endemic countries to use condom or to avoid sexual intercourse and, in any case, to avoid blood and semen donation for at least 6 months [14, 15]. As the epidemic will continue to spread, and more cases will be reported, it is possible that some unexpected clinical findings will emerge and increasing evidences will contribute to drive the correct indications for the patients.

The dissemination of appropriate knowledge and diagnostic capability about ZIKV is important in Italy, where the competent vector is present [7, 16]. In fact, in Italy *Aedes* mosquitoes sustained an autochthonous outbreak of CHIKV, with 205 cases in 2007.

## Additional file

Additional file 1: File name: “Three cases of Zika – CARE checklist”; Title of data: CARE checklist for Case Report; Description of data: a file describing the adherence of the manuscript to CARE checklist, with reference of page for each item. (DOC 48 kb)

## Abbreviations

CHIKV: Chikungunya
INMI: National Institute for Infectious Diseases
PCR: Polymerase chain reaction
ZIKV: Zika Virus
