# Supplementary material for: Three cases of Zika virus imported in Italy: need for a clinical awareness and evidence-based knowledge
Source: BMC Infect Dis. 2016 Nov 11;16:669. doi: 10.1186/s12879-016-1973-5 (PMC5106818; doi:10.1186/s12879-016-1973-5)
Supplement: Additional file 1: — File name: “Three cases of Zika – CARE checklist”; Title of data: CARE checklist for Case Report; Description of data: a file describing the adherence of the manuscript to CARE checklist, with reference of page for each item. (DOC 48 kb) [file 12879_2016_1973_MOESM1_ESM.doc]

## CARE CHECKLIST of information to include when writing a case report

| **Section** | **Item** | **Checklist item description** | **Reported on page** |
| --- | --- | --- | --- |
| **Title** | 1 | The words “case report” and the area of focus should appear in the title (such as diabetes, a therapeutic approach, an outcome) | 1 |
| **Key Words** | 2 | 2 to 5 key words that identify areas covered in this case report | 3 |
| **Abstract** | 3a | Introduction—What is unique about this case? What does it add to the medical literature? Why is this important? | 3 |
|  | 3b | The patient's main concerns and important clinical findings | 3 |
|  | 3c | The main diagnoses, therapeutics interventions, and outcomes | 3 |
|  | 3d | Conclusion—What are the “take-away” lessons from this case? | 3 |
| **Introduction** | 4 | One or two paragraphs summarizing why this case is unique with reference to the relevant medical literature | 4 |
| **Patient Information** | 5a | De-identified demographic and other patient specific information | 4-6 |
|  | 5b | Main concerns and symptoms of the patient | 4-6 |
|  | 5c | Medical, family, and psychosocial history including relevant genetic information (this should also appear in the timeline) | - |
|  | 5d | Relevant past interventions and their outcomes | - |
| **Clinical Findings** | 6 | Describe the relevant physical examination (PE) and other significant clinical findings | 4-6 |
| **Timeline** | 7 | Relevant data from the patient's history organized as a timeline | 4-6 |
| **Diagnostic Assessment** | 8a | Diagnostic methods (PE, laboratory testing, imaging, surveys) | 5-6 |
|  | 8b | Diagnostic challenges (access, financial, cultural) | 6 |
|  | 8c | Diagnostic reasoning including other diagnoses considered | 4-6 |
|  | 8d | Prognostic characteristics when applicable (staging) | 4-6 |
| **Therapeutic Intervention** | 9a | Types of intervention (pharmacologic, surgical, preventive) | - |
|  | 9b | Administration of intervention (dosage, strength, duration) | - |
|  | 9c | Any changes in the interventions (with rationale) | - |
| **Follow-up and Outcomes** | 10a | Clinician and patient-assessed outcomes (when appropriate) | 4-6 |
|  | 10b | Important follow-up diagnostic and other test results | 4-6 |
|  | 10c | Intervention adherence and tolerability (how was this assessed) | - |
|  | 10d | Adverse and unanticipated events | - |
| **Discussion** | 11a | Strengths and limitations in your approach to this case | - |
|  | 11b | Discussion of the relevant medical literature | 6 |
|  | 11c | The rationale for your conclusions (a causality assessment) | 6 |
|  | 11d | The primary “take-away” lessons from this case report | 6 |
| **Patient Perspective** | 12 | When appropriate the patient should share their perspective on the treatments they received | - |
| **Informed Consent** | 13 | Did the patient give informed consent? | Yes |
